# Dysmenorrhea severity in war refugees with hypertension: a cross-talk with antihypertensives and analgesics

**DOI:** 10.4314/ahs.v23i4.47

**Published:** 2023-12

**Authors:** Omar Salem Gammoh

**Affiliations:** Department of Clinical Pharmacy and Pharmacy Practice, Faculty of Pharmacy, Yarmouk University -Irbid, Jordan

**Keywords:** Abdominal pain, hypertension, menstruation disorder

## Abstract

**Background:**

Dysmenorrhea is the most common gynecological condition among women of reproductive age. Investigating the cross-talk between hypertension and dysmenorrhea is attractive and understudied, moreover, menstrual health is poorly studied in refugees.

**Objectives:**

The present study aims at examining the association between dysmenorrhea severity and antihypertensives and analgesics namely acetaminophen and Non-Steroidal Anti-inflammatory Drugs (NSAIDs) used by traumatized Syrian war refugees with hypertension

**Methods:**

This is a cross-sectional study recruiting Syrian female refugees with hypertension. A structured questionnaire probed their demographics and clinical data. Dysmenorrhea severity was assessed using the WaLIDD (working ability, location, intensity, days of pain, dysmenorrhea) self-report scale

**Results:**

Data were analysed from 125 patients, almost half were diabetic, 47 (37.6%) had dyslipidemia, 59 (47%) reported using β-blockers, 56 (44.8%) reported using ACEIs/ARBs, 43 (34.2) reported using CCBs and 30 (25%) were using diuretics. According to the multivariate binary logistic regression, severe dysmenorrhea was associated with acetaminophen OR 6.5, 95%CI (1.39–30.55), p=0.02 and NSAIDs use OR 2.97, 95%CI (1.28–6.89), p=0.02. Antihypertensive drugs were not associated with dysmenorrhea severity.

**Conclusion:**

Determinants of severe dysmenorrhea in patients with hypertension need more study, herein we report that analgesics but not antihypertensives are not associated with dysmenorrhea severity.

## Introduction

Dysmenorrhea is the most common gynecological condition among women of reproductive age 1 characterized by severe abdominal pain and has a considerable impact on quality of life 2. Dysmenorrhea is relatively understudied and poorly managed in practice despite the availability of analgesics such as acetaminophen and Non-Steroidal Anti-inflammatory drugs (NSAIDs) 3,4 Menstrual health research among refugee women is still emerging; however, there is evidence that dysmenorrhea is common among displaced refugee women 5. A recent study found a dysmenorrhea prevalence rate of over 96% among Palestinian adolescent refugees living in camps in the West Bank and Jordan 6.

Hypertension is the most frequent non-communicable disease among Syrian refugees 7 and is associated with serious cardiovascular complications 8,9 Women are at higher risk for complications and mortality compared to males 10.

Investigating the association between hypertension and dysmenorrhea is attractive and requires more studies. Hypertension and dysmenorrhea have mutual risk factors such as weight, age, and smoking 11. Furthermore, hypertension and dysmenorrhea overlap in inflammatory regulatory pathways involving increased prostaglandin-related inflammatory biomarkers production.^12^,^13^ Moreover, the hormones that mediate the menstrual cycle may interfere with hypertension. For instance, estrogen induces vasodilation 14,15 while androgen is associated with hypertension pathogenesis 16

The anti-inflammatory effects of different antihypertensive agents are documented. For example, β-blockers have anti-inflammatory effects through the reduction release of IL-6 and TNF-α 17,18, furthermore, angiotensin II is implicated in pro-nociceptive pain signaling and its inhibition represents a novel pain-reducing strategy 19 and the calcium channels activation play an important role in hence nociceptive signaling at all levels from the periphery to the brain 20.

Studying the determinants of severe dysmenorrhea in refugees with hypertension is crucial because identifies factors associated with dysmenorrhea severity hence this can improve the menstrual cycle experience and the quality of life of this vulnerable group. Although our research group investigated determinants of severe dysmenorrhea in refugees' communities, however, we did not investigate determinants of severe dysmenorrhea in refugees with hypertension.

War-displaced refugees with chronic diseases experience high inflammatory status 21.

The present research tried to answer the following questions: Based on their postulated anti-inflammatory effects, what is the possible association of antihypertensives with dysmenorrhea symptoms in refugees with hypertension? Also, are the dysmenorrhea over-the-counter analgesics namely acetaminophen and NSAIDs associated with dysmenorrhea severity?

Therefore; the present study aims at examining the association between dysmenorrhea severity and antihypertensives and analgesics used by traumatized Syrian war refugees with hypertension.

## Methods

### Study design and settings

This is a cross-sectional cohort study approved by the IRB Committee at Yarmouk University and the Caritas-Jordan, a catholic not-for-profit organization. The medical charts for female hypertensive patients visiting the primary care clinics in the urban districts of Jordan were extracted. A female research assistant approached the participants via phone calls to explain the study objective and methods. Afterward, the link containing the structures study instrument was sent to the interested females where the first step was to read and sign the consent form electronically and then to enrol in the study voluntarily. All participants had the right to withdraw from the study at any time. The research assistant assisted illiterate participants by reading out the study questionnaire over the phone.

### Inclusion criteria

Participants were recruited based on the following inclusion criteria: diagnosed with hypertensive as per the Joint National Committee (JNC 8) guidelines 22 for at least one year; residing in Jordan for at least 5 years; received primary education, unemployed; non-smokers, using only acetaminophen or NSAIDs for dysmenorrhea symptoms and adhering to their antihypertensive medications according to the Arabic-validated version of Morisky scale 23,24 The scale is composed of 4 items which are behaviors associated with medication non-adherence, e.g., “do you forget to take your medication?” with simple scoring options as yes and no. The Arabic translation was demonstrated to be reliable with a Cronbach's alpha score of 0.82 24. A score of 3 or less out of 4 reflects moderate adherence.

### Study instrument

#### Covariates

A self-administered structured online questionnaire was employed to cover the participants' demographical and clinical data including age, marital status, the number of years of displacement in Jordan, current residence location, and the presence of diabetes and dyslipidemia.

#### Exposure

To record the antihypertensive medications used by the patients, the approved list of antihypertensive medications dispensed at Caritas pharmacy was included in a checklist on the online questionnaire in addition to the information presented in the medical charts. To ensure the accuracy of data collection, both the generic name, the brand name, and the picture of the medication pack were all presented and checked out by the patients. The antihypertensive classes used were β-blockers (bisoprolol, atenolol), Diuretics (hydrochlorothiazide), Calcium Channel Blockers (CCBs) (amlodipine), Angiotensinogen Converting Enzyme Inhibitors (ACEIs) (enalapril) and Angiotensinogen Receptor Blockers (ARBs) (valsartan). All doses of the medications were within the therapeutic range.

Regarding the over-the-counter analgesics all the available generic names for acetaminophen or NSAIDs were included, participants reporting other pain-relief methods were excluded.

#### Dysmenorrhea

To evaluate the severity of dysmenorrhea, the WaLIDD (working ability, location, intensity, days of pain, dysmenorrhea) self-report scale was used 25. The WaLIDD scale comprises four subscales that measure features of dysmenorrhea, specifically: 1) the number of anatomical pain locations (no part of the body, lower abdomen, lumbar region, lower limbs, inguinal region); 2) pain range (does not hurt, hurts a little, hurts a little more, hurts even more, hurts a lot, hurts a lot more); 3) the number of days of pain during menstruation (0, 1–2 days, 3–4 days, and gt;5 days); and 4) the frequency of disabling pain impacting ability to perform their activities (never, rarely, almost always, always). Each subscale provided a specific score between 0 and 3, and the total score ranged from 0 to 12 points. The score interpretations are as follows: 0 without dysmenorrhea, 1–4 mild dysmenorrhea, 5–7 moderate dysmenorrhea, and 8–12 severe dysmenorrhea.

## Results

### Response rate

A total of 181 patients were approached, 56 patients were excluded as they did not completely fit the inclusion criteria, and declined participation. Therefore; the response rate was 69%.

### Participants' demographics and clinical data

Data were analysed from 125 patients, 84 (67.2%) were above 41 years old, 83 (66.4%) were married, 85 (68%) were displaced for a period between 5-10 years, almost half were diabetic, 47 (37.6%) had dyslipidemia, 59 (47%) reported using β-blockers, 56 (44.8%) reported using ACEIs/ARBs, 43 (34.2) reported using CCBs and 30 (25%) were using diuretics. Table 1.

**Table 1:** Respondents' characteristics

Factor	Category	n (%)
Age	Below 40	41 (32.8)
	41 and above	84 (67.2)
Marital status	Single	42 (33.6)
	Married	83 (66.4)
Years of displacement	5-10 years	85 (68)
	>10 years	40 (32)
Diabetes	No	56 (44.8)
	Yes	69 (55.2)
Dsylipidemia	No	78 (62.4)
	Yes	47 (37.6)
β-blockers	No	65 (53)
	Yes	59 (47)
ACEIs/ARBs	No	67 (53.6)
	Yes	56 (44.8)
CCBs	No	81 (64.8)
	Yes	43 (34.2)
Diuretics	No	94 (75)
	Yes	30 (25)
Acetaminophen	No	23 (18.4)
	Yes	102 (81.6)
NSAIDs	No	81 (64.8)
	Yes	43 (34.2)

### Dysmenorrhea severity

According to WaLIDD scale, 88 (70.4%) patients did not meet the threshold score for severe dysmenorrhea while 37 (29.6%) reported a score of 8 or higher indicating severe dysmenorrhea.

### Factors associated with severe dysmenorrhea

The chi-square analysis revealed that all the antihyper-tensive classes were not associated (p>0.05) with severe dysmenorrhea, however, the use of acetaminophen and NSAIDs was significantly associated(p<0.05) with severe dysmenorrhea. Table 2.

**Table 2:** Association between the patients factor and dysmenorrhea severity

Factor	Category	Non-severe dysmenorrhea	Severe dysmenorrhea	p-value
Age	Below 40	29 (33)	12 (32.4)	0.57
	41 and above	59 (67)	25 (67.6)	
Marital Status	Single	31 (35.2)	11 (29.7)	0.35
	Married	57 (68.8)	26 (70.3)	
Years of displacement	5-10 years	61 (69.3)	24 (64.9)	0.39
	>10 years	27 (30.7)	13 (35.1)	
Diabetes	No	39 (44.3)	17 (45.9)	0.51
	Yes	49 (55.7)	20 (54.1)	
Dyslipidemia	No	52 (59.1)	26 (70.3)	0.17
	Yes	36 (40.9)	11 (29.7)	
β-blockers	No	46 (52.3)	19 (52.8)	0.56
	Yes	42 (47.7)	17 (47.2)	
ACEIs/ARBs	No	46 (52.3)	21 (58.3)	0.22
	Yes	42 (47.7)	14 (38.9)	
CCBs	No	60 (68.2)	21 (58.3)	0.20
	Yes	28 (31.8)	15 (41.7)	
Diuretics	No	68 (77.3)	26 (72.2)	0.35
	Yes	20 (22.7)	10 (27.8)	
Acetaminophen	No	21 (23.9)	2 (5.4)	0.01*
	Yes	67 (76.1)	35 (94.6)	
NSAIDs	No	63 (71.6)	18 (50)	0.02*
	Yes	25 (28.4)	18 (50)	

A chi-square test was used to investigate the association between the patients' factors and dysmenorrhea severity. Dysmenorrhea severity was measured using WALLiD scale, a score above 7 was used as a cut-off value to indicate severe dysmenorrhea. β-blockers: Beta blockers, ACEIs: Angiotensin Converting Enzyme Inhibitors, ARBs: Angiotensin II Receptor Blockers, CCBs: calcium Channel Blockers, NSAIDs: Non-steroidal anti-inflammatory drugs

According to the multivariate binary logistic regression model with dysmenorrhea severity as the dependent variable and adjusted for “using acetaminophen” and “using NSAIDs” showed that patients using acetaminophen were at higher risk OR 6.5, 95%CI (1.39-30.55), p=0.02 for severe dysmenorrhea, also patients using NSAIDs were at higher risk OR 2.97, 95%CI (1.28-6.89), p=0.02 for severe dysmenorrhea. Table 3

**Table 3:** Predictors of severe dysmenorrhea

Factor	B	wald	OR	95% CI	p-value
Using Acetaminophen	1.87	5.66	6.52	1.39-30.55	0.02
Using NSAIDs	1.09	6.45	2.97	1.28-6.89	0.01

Multivariate binary logistic regression was used to investigate the independent variables and the dependent variable (dysmenorrhea severity). Dysmenorrhea severity was measured using WALLiD scale, a score above 7 was used as a cut-off value to indicate severe dysmenorrhea. β-blockers: Beta blockers, ACEIs: Angiotensin Converting Enzyme Inhibitors, ARBs: Angiotensin II Receptor Blockers, CCBs: Calcium Channel Blockers, NSAIDs: Non-steroidal anti-inflammatory drugs

## Discussion

The present study aims at examining whether a specific antihypertensive class(s) and analgesics could be associated with less dysmenorrhea severity in a cohort of displaced war refugees with hypertension. We report that β-blockers, ACEIs/ARBs, CCBs, and diuretics were not associated with dysmenorrhea severity, however, the use of acetaminophen and NSAIDs was associated with severe dysmenorrhea.

The anti-inflammatory/antinociceptive effects of antihypertensive agents are rarely studied. This work aimed at examining the potential benefits of these medications in a group of traumatized refugees. It is established that refugees suffer from high psychological stress and chronic diseases associated with high inflammatory status 21 that could be related in part to pain sensitivity 26. While some evidence pointed out the anti-inflammatory/antinociceptive effects of antihypertensive medications however this was not reflected in dysmenorrhea severity, perhaps because the study sample was “challenging” for these medications to exert a significant effect.

Our findings revealed that the use of acetaminophen and NSAIDs, the most common OTC self-medications for dysmenorrhea were associated with dysmenorrhea severity. This association can be explained as follows, women with severe dysmenorrhea were more likely to use analgesics (27). However, despite using acetaminophen and NSAIDs, dysmenorrhea severity is not alleviated and this indicates the need for additional management strategies (28,29) to control dysmenorrhea severity in this population. The regression model examines the association between the dependent variable (dysmenorrhea severity) with the independent variables rather than a causal relationship.

This is the first study that tries to investigate the association between antihypertensive agents and dysmenorrhea severity in traumatized refugees. The novelty, the strict inclusion criteria, and the validated scales are strengths, on the other hand, the cross-sectional design and the limited sample size for each class of medication are limitations. Furthermore, other factors that could be associated with severe dysmenorrhea can be studied in the future such as the number of pregnancies and other gynecological disorders.

In conclusion, apparently, antihypertensives do not worsen dysmenorrhea severity, future studies with higher sample size and longitudinal design are required.
